# Foreign

**Published:** 1841-03-13

**Authors:** 


### FOREIGN.

*On the Microscopic Constituents of Milk.* By Professor NASSE, of Marburg.—After a careful microscopic examination of milk from pregnant and suckling women, as well as from a cow and a bitch, and a comparison of his results with those of Donné and other preceding observers, the author says that the following may be enumerated as the microscopic constituents of the normal secretion of the mammary gland: 1. The smooth, homogeneous, transparent oil-globules, to which, in addition to the common milk-globules, belong also the fine, scarcely measurable particles and the larger drops of oil which swim on the top of the milk. 2. The cream-globules which are distinguished from the oil-globules by their opacity and their facette-like aspect. 3. The granulated yellow corpuscles. 4. The lamella of epithelium. 5. The more or less turbid medium, in which the four preceding kinds of corpuscles are suspended.

The first, the common milk-globules, are composed entirely of fatty matter, which dissolves completely and rapidly in ether. No membrane can be seen investing them. In the first nine days after delivery, the largest globules measure 1-200th of a line in diameter, afterwards they are as much as 1-100th, but many are found of a much smaller size, and through all periods of lactation, the microscope, as well as other means of examination, show that the proportion of oil-globules in the milk varies greatly in different persons and under different circumstances.

In perfectly fresh warm woman's milk, no other globules than these are sometimes found. But as soon as the milk has stood for some time exposed to the air, other corpuscles are discernible in it which are distinguished from the preceding by a greater definiteness, a less degree of polish, and an appearance of facettes. In size they are nearly similar to the oil-globules, but if the milk be examined some time after it is drawn a number are found much larger; 1-50th of a line or even more in diameter. They are not so easily soluble in ether as the common milk-globules; they do not break up in drying, but they become clearer; acetic acid and ammonia have no influence upon them; they diminish for a time when the milk is boiled, but they re-appear gradually as it cools again; when left at rest they collect on the surface and form the cream; they easily stick together, and butter is formed when they are collected in one homogeneous mass. It is evident that they acquire their peculiar characters after they are drawn from the gland-ducts, for the author, as he watched them on the field of the microscope, could see individual globules which were originally clear, becoming on a sudden quite dark, and assuming the several characters of the cream-globules.

The yellow granulated corpuscles are almost peculiar to the colostrum; after the first few days from delivery, they cease to occur in the milk, and they disappear from it earlier in those who have borne several children than in primiparæ. They are not all spherical; the majority are flat. Their diameter is at most from 1-200th to 1-100th of a line; some are found measuring 1-50th in length and 1-75th in breadth. They consist of small clear globules of fatty matter, which are connected together by a firm cement which is unalterable by either ammonia or concentrated acetic acid, or by boiling. When the milk is left at rest, these globules collect on its surface; and when they exist in considerable numbers render it unfit for making butter. The author believes that they are not, like the preceding globules, formed by the action of the air, but that they are produced by the secreting surface of the gland-ducts, and are analogous to the mucus-cells which are cast off from the surfaces of many mucous membranes, and to which they are in many respects very similar.—*Brit. and For. Med. Rev.*, *from Müller's Archiv.*, 1840. Heft iii., p. 258.

*Effects of Over-eating.* By Dr. Ritter.—A girl, three years old, went with her father to gather plums; as they fell from the trees she kept eating an enormous quantity of them, and then, feeling very thirsty, drank cold water from a spring close by. Soon after returning home, having seemed previously quite well, she suddenly fell down as if struck dead by lightning; her eyes were convulsively pulled about, and she lay quite senseless. The author being immediately sent for found her with her eyes staring wildly, unconscious, insensible, and speechless; her pulse was suppressed, scarcely perceptible, and small; her respiration short and quick, frequently interrupted by sighing, and her face pale. The precordial region was distended and very tense. An emetic, containing one-eighth of a drachm of vinum antimonii, and one-eighth of a grain of tartar emetic, was immediately given, and was repeated every five minutes, but after several doses produced no effect. Three powders were then given at intervals of ten minutes, each of which contained a grain of tartar emetic, and six grains of ipecacuanha, but these excited only retching; vomiting was at last brought on by tickling the pharynx with a feather. The materials discharged consisted of a huge mass of tough, half-digested plums, with some frothy fluid. The severest convulsions now ensued, especially on the right side of the body. The eyes rolled irregularly and convulsively in their orbits; the mouth was drawn over to the right side, and watery froth kept running from it; the right arm and foot were set into a constant swinging motion, the face was earthy, the glance oblique, and the nose pointed; in short, there were all the appearances of closely impending death. Some strong coffee, and some wine and water were given, but the convulsions still continued. A mixture of equal parts of castor oil and almond oil was next prescribed, and a spoonful of it was given every ten minutes; clysters, containing oil, vinegar, and salt, were also administered. Soon after the second of these, borborygmi commenced, and were presently followed by the discharge of an enormous mass of half-digested plums, which was again repeated for several times. The child not long after came to herself again, and though weak was still free from apparent danger. She slept well during the night, and next morning, with the exception of dulness and repeated diarrhœa, was well and in good spirits.—*Ibid.*, *from Medicinische Zeitung.*

